# (K, Na)NbO_3_-based lead-free piezoceramics: one more step to boost applications

**DOI:** 10.1093/nsr/nwac101

**Published:** 2022-05-31

**Authors:** Huan Liu, Yi-Xuan Liu, Aizhen Song, Qian Li, Yang Yin, Fang-Zhou Yao, Ke Wang, Wen Gong, Bo-Ping Zhang, Jing-Feng Li

**Affiliations:** State Key Laboratory of New Ceramics and Fine Processing, School of Materials Science and Engineering, Tsinghua University, China; School of Materials Science and Engineering, University of Science and Technology Beijing, China; State Key Laboratory of New Ceramics and Fine Processing, School of Materials Science and Engineering, Tsinghua University, China; State Key Laboratory of New Ceramics and Fine Processing, School of Materials Science and Engineering, Tsinghua University, China; School of Materials Science and Engineering, University of Science and Technology Beijing, China; State Key Laboratory of New Ceramics and Fine Processing, School of Materials Science and Engineering, Tsinghua University, China; School of Materials Science and Engineering, University of Science and Technology Beijing, China; Center of Advanced Ceramic Materials and Devices, Yangtze Delta Region Institute of Tsinghua University, China; State Key Laboratory of New Ceramics and Fine Processing, School of Materials Science and Engineering, Tsinghua University, China; Center of Advanced Ceramic Materials and Devices, Yangtze Delta Region Institute of Tsinghua University, China; School of Materials Science and Engineering, University of Science and Technology Beijing, China; State Key Laboratory of New Ceramics and Fine Processing, School of Materials Science and Engineering, Tsinghua University, China

Piezoelectric materials, as a conversion medium between mechanical and electrical energies, are widely used in actuators, sensors and sonar systems. Lead-based materials represented by lead zirconate titanate (PZT) have dominated the market for decades due to their exceptional performance. Over the past 20 years or so, the ever-increasing awareness of the need for environmental protection has driven the research and development of environmentally friendly lead-free piezoceramics [1,2]. Among lead-free piezoelectric systems, (K, Na)NbO_3_ (KNN) has been revealed as the most promising candidate for lead-free piezoelectric applications.

The active research on KNN was stimulated by a paper in *Nature* in 2004 by Saito *et al*. [3]. Since then, significant research progress has been achieved in the development of high-performance KNN-based ceramics. The piezoelectricity enhancement was realized mainly by phase boundary engineering. Coexisting phases with comparable free energies, such as rhombohedral-tetragonal (*R-T*) and rhombohedral-orthorhombic-tetragonal (*R-O-T*) phases, can be obtained around ambient temperature via chemical modification, to enhance the piezoelectric properties of KNN-based materials [4]. An exceptional peak *d*_33_ of 704 pC/N was achieved in a textured (K, Na)(Nb, Sb)O_3_-CaZrO_3_-(Bi, K)HfO_3_ composition in 2018 [5]. Recently, Wu *et al*. constructed a nanoscale *R-O-T* multiphase coexistence system through multi-element doping and obtained an ultrahigh *d*_33_ of 650 ± 20 pC/N in non-textured KNN-based ceramics, which is superior to commercial PZT-5H (*d*_33_ = 620 pC/N) [6]. Excellent piezoelectric properties (*d*_33_ > 500 pC/N) can also be obtained by optimizing lattice distortion [7]. As shown in Fig. S1, the *d*_33_ of KNN-based ceramics has compared with or surpassed commercial PZT after decades of research.

Despite a lot of research into the KNN system, a major problem remains that has long prevented it from replacing lead-based counterparts. Namely, more attention should be paid to another important property, mechanical quality factor (*Q*_m_), which is a parameter indicating the capacity to mitigate the energy loss during electromechanical interconversion. For most applications, a combination of high *d*_33_ and moderate *Q*_m_ is preferred; especially in resonant devices, so-called hard-type piezoceramics with high *Q*_m_ are prerequisite. Figure 1a shows a comparison of the representative figures of merit (i.e. *Q*_m_ and *d*_33_) between KNN-based and PZT-based materials. It is clear from the *Q*_m_-*d*_33_ projection plane that the *d*_33_ values of state-of-the-art soft-type KNN-based ceramics are level-pegging with commercial PZT (450–650 pC/N).

**Figure 1.**
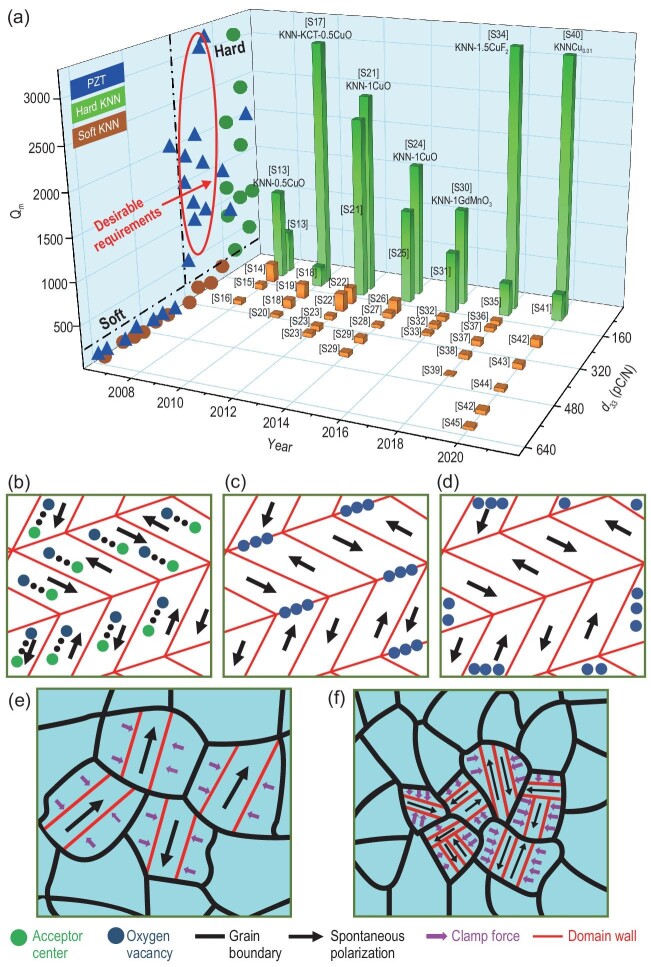
(a) *d*_33_ and *Q*_m_ of KNN-based and PZT-based piezoceramics. All references in Fig. 1a can be found in Table S1. (b–d) Defect engineering. (e and f) Grain-size engineering for boosting *Q*_m_ of KNN-based materials.

However, the comprehensive performance of hard-type KNN-based ceramics is far inferior to PZT. Although the *Q*_m_ of hard-type KNN-based materials can reach a high level of 2000, there is a distinct gap between KNN- and PZT-based materials in the aspect of *d*_33_ values. Simultaneously, high *Q*_m_ and desirable *d*_33_ (e.g. ∼250 pC/N) are demanded for high-intensity focused ultrasound applications. The satisfactory comprehensive properties (*Q*_m_ and *d*_33_) sustain the dominance of lead-based materials in the market. Notably, remarkable enhancement of *Q*_m_ (usually >800) is usually accompanied by a striking deterioration of *d*_33_ (decreasing to ∼160 pC/N) in existing KNN systems, which is a serious disadvantage for high-power applications. Therefore, the *Q*_m_ and *d*_33_ of hard-type KNN-based materials must be co-engineered to broaden the application range.

Theoretically, *Q*_m_ is closely related to domain-wall motions and the corresponding hardening mechanisms have been well conceptualized in lead-based materials. Figure S2a and b shows the pinning energies of various defects or defect dipoles and the barrier energy for the movement of domain walls in lead-based systems respectively [8]. Defects can inhibit domain-wall motion, leading to enhanced *Q*_m_. Therefore, acceptor doping is the main scheme adopted for hardening. Recently, a new strategy has been proposed to achieve both high *Q*_m_ and desirable *d*_33_ in PZT-based ceramics by synergistically adjusting the defect structure and domain-wall density [9]. It is believed that this strategy can also be applied to KNN-based materials to solve the long-standing dilemma.

Concerning the KNN system, three models derived from the lead-based system are instructive for hardening, including bulk effect, domain-wall effect and surface effect, as illustrated in Fig. 1b–d. For the bulk effect, the localization of oxygen vacancies around the acceptor centers has been experimentally observed and the defect dipoles are aligned parallel to the direction of spontaneous polarization. Therefore, there is a strong restriction for the domain-wall motion. Lattice defects tend to migrate gradually towards domain walls over time due to lower configurational energies. Aggregation of defects along domain walls would create a strong pinning force to the domain-wall motion and consequently lead to a hardening effect, which is the principle of the domain-wall effect. In contrast to the domain-wall effect, a long-range diffusion of mobile charged carriers towards the surface boundary is considered to be the surface effect. Inspired by these models, many studies have been launched to harden KNN-based piezoelectric ceramics. A simultaneous increase in *Q*_m_ and *d*_33_ is also reported in the KNN-based ceramics containing compounds such as K_4_CuNb_8_O_23_, MnO_2_ and Co_2_O_3_ (Table S2) [10]. In addition, there exists another feasible way to enhance *Q*_m_ by refining grains. As illustrated in Fig. 1e and f, grain boundaries also play an important role in clamping the domain wall. Therefore, large *Q*_m_ may be obtained by grain engineering.

The application of soft-type KNN-based ceramics has made great contributions to the development of lead-free piezoelectrics. From the above discussion, we note that hard-type KNN-based materials need to meet the requirements of high-power applications. We believe that this challenging issue will certainly encourage research activities that are devoted to improving the *Q*_m_ with suitable *d*_33_. The target of future research should be that *Q*_m_ breaks the barrier of 500 and *d*_33_ reaches ∼300 pC/N, which are comparable to the performance of commercial hard-type PZT-based materials. The combined use of defect engineering, grain-size engineering and domain engineering can be an effective strategy. In addition, other pivotal parameters including dielectric loss (<0.004), longitudinal electromechanical coupling factor *k*_33_ (>0.6), Curie temperature *T*_C_ (>280°C) and dielectric constant (>1000) should also be considered in high-power applications. It is expected that large-scale, high-power applications of lead-free KNN piezoceramics will be further accelerated, especially if breakthroughs in the synergistic improvement of *Q*_m_ and *d*_33_ are made.

## Supplementary Material

nwac101_Supplemental_File
