# Supplementary material for: (K, Na)NbO3-based lead-free piezoceramics: one more step to boost applications
Source: Natl Sci Rev. 2022 May 31;9(8):nwac101. doi: 10.1093/nsr/nwac101 (PMC9385458; doi:10.1093/nsr/nwac101)
Supplement: nwac101_Supplemental_File [file nwac101_supplemental_file.docx]

**(K, Na)NbO_3_-based lead-free piezoceramics: one more step to boost applications**

Huan Liu^2,1#^, Yi-Xuan Liu^1#^, Aizhen Song^2,1^, Qian Li^1^, Yang Yin^2^, Fang-Zhou Yao^3^, Ke Wang^1^, Wen Gong^3^, Bo-Ping Zhang^2^*, Jing-Feng Li^1^*

^1^ State Key Laboratory of New Ceramics and Fine Processing, School of Materials Science and Engineering, Tsinghua University, China.

^2^ School of Materials Science and Engineering, University of Science and Technology Beijing, China.

^3^ Center of Advanced Ceramic Materials and Devices, Yangtze Delta Region Institute of Tsinghua University, China

***Corresponding author.** E-mail: bpzhang@ustb.edu.cn; jingfeng@mail.tsinghua.edu.cn

**#**Equally contributed to this work.





**Figure S1.** Historical evolution in *d*_33_ of KNN-based ceramics as a function of time measured in years. All *d*_33_ values were recorded at room temperature and the blue triangles are the representative PZT-based materials [1-11].


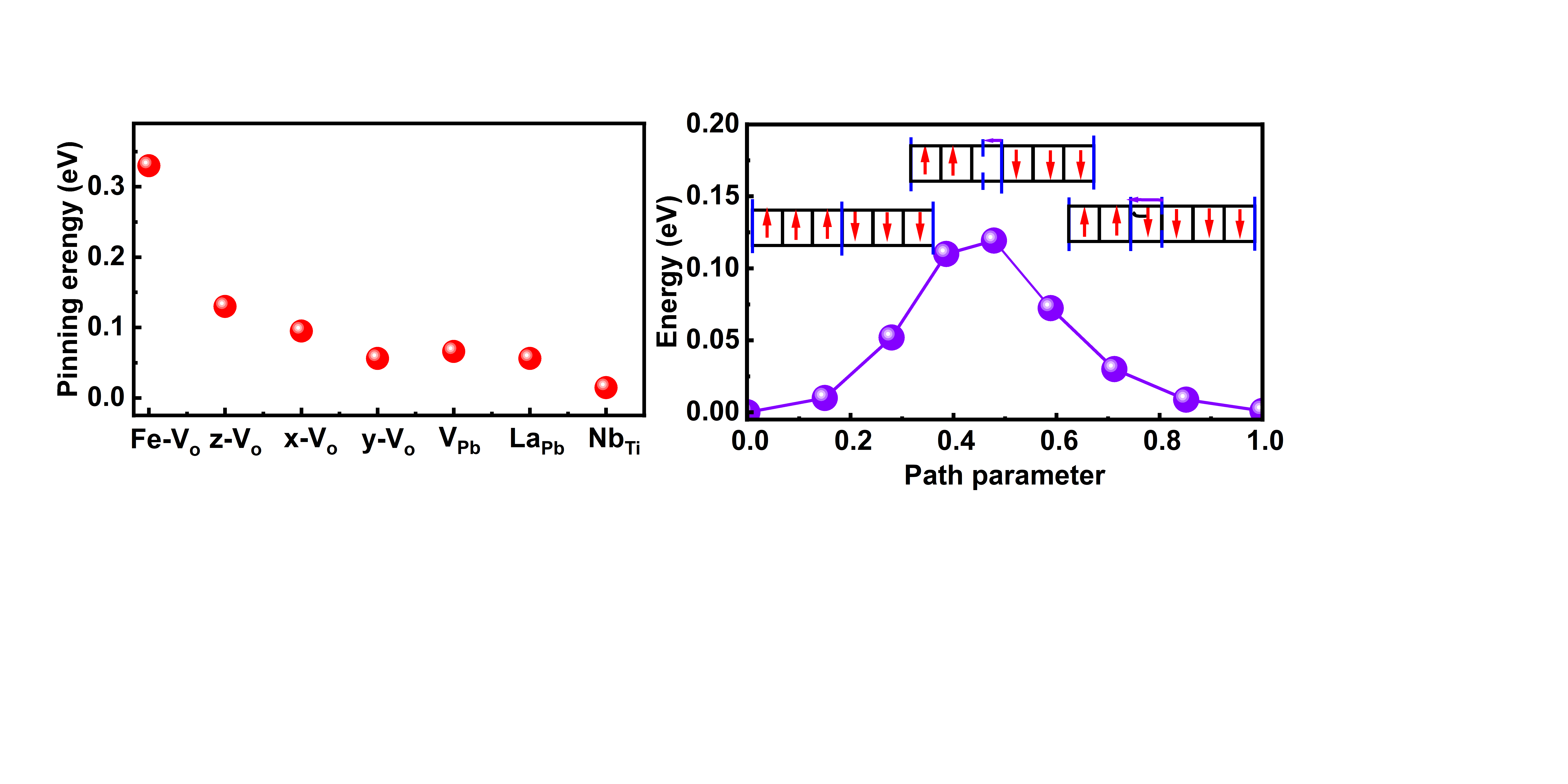


**Figure S2.** (a) Pinning strength of various defects in lead-based materials. (b) The barrier energy for the movement of the domain wall across a unit cell. (a)-(b) Reproduced with permission from ref [12]. Copyright 2013, American Physical Society.

**Table S1.** The performance of KNN-based and PZT-based materials

| Type | Composition | *d*_33_  (pC/N) | *Q*_m_ | Year | Reference |
| --- | --- | --- | --- | --- | --- |
| PZT-based  materials | PZT | 58 | 2000 | 2021 | -- |
|  | PZT | 130 | 830 | 2021 | -- |
|  | PIC300 | 155 | 1400 | -- | [11] |
|  | PZT | 200 | 3159 | 2021 | -- |
|  | PZT | 226 | 1600 | 2021 | -- |
|  | PZT-8 | 230 | 1000 | -- | [11] |
|  | PZT | 232 | 3065 | 2021 | -- |
|  | PIC181 | 265 | 2000 | -- | [11] |
|  | PZT | 271 | 970 | 2021 | -- |
|  | PIC241 | 290 | 1200 | -- | [11] |
|  | PZT | 296 | 1800 | 2021 | -- |
|  | PZT-4 | 300 | 500 | -- | [11] |
|  | PZT | 302 | 1500 | 2021 | -- |
|  | PZT | 350 | 1947 | 2021 | -- |
|  | PZT-5A | 374 | 80 | -- | [11] |
|  | PZT | 410 | 80 | 2021 | -- |
|  | PZT | 454 | 87 | 2021 | -- |
|  | PIC151 | 500 | 100 | -- | [11] |
|  | PZT | 556 | 55 | 2021 | -- |
|  | PZT | 603 | 70 | 2021 | -- |
|  | PZT | 635 | 70 | 2021 | -- |
| KNN-based  materials | KNNL-0.45CuO | 64 | 523 | 2008 | [13] |
|  | KNN-0.45CuO | 97 | 1111 | 2008 | [13] |
|  | KNN-Mn-0.3LiCO_3_ | 136 | 234 | 2008 | [14] |
|  | KNNS6 | 186 | 71 | 2008 | [15] |
|  | KNN-LSN-0.2Ag_2_O | 278 | 55 | 2008 | [16] |
|  | KNN-KCT-0.5Cu | 94 | 3053 | 2010 | [17] |
|  | KNN-1Ba(Ti_0.9_Sn_0.1_)O_3_ | 120 | 251 | 2010 | [18] |
|  | KNN-(Bi_0.5_K_0.5_)TiO_3_-0.4MnO | 196 | 183 | 2010 | [19] |
|  | KNN-6Ba(Ti_0.9_Sn_0.1_)O_3_ | 257 | 111 | 2010 | [18] |
|  | KNN-LS-0.1FeO | 311 | 45 | 2010 | [20] |
|  | KNNCu1 | 96 | 2542 | 2012 | [21] |
|  | KNNZn0.25 | 132 | 2217 | 2012 | [21] |
|  | LKNN | 186 | 206 | 2012 | [22] |
|  | LKNN | 229 | 220 | 2012 | [22] |
|  | KNN-BZ-5(Bi_0.5_K_0.5_)TiO_3_ | 279 | 53 | 2012 | [23] |
|  | KNN-BZ-5(Bi_0.5_K_0.5_)TiO_3_  -Mn | 339 | 65 | 2012 | [23] |
|  | KNN-BZ-6(Bi_0.5_K_0.5_)TiO_3_  -Mn | 372 | 52 | 2012 | [23] |
|  | KNN-Cu | 82 | 1633 | 2014 | [24] |
|  | LKNNT-3Cu | 129 | 1148 | 2014 | [25] |
|  | KNN-6LN | 197 | 159 | 2014 | [26] |
|  | KNNL3-(Bi_0.5_K_0.5_)TiO_3_-BZ | 227 | 69 | 2014 | [27] |
|  | KNN-LS-BiFeO_3_-1.5Zn | 280 | 41 | 2014 | [28] |
|  | KNNS-Bi_0.5_(Na_0.82_K_0.18_)_0.5_ZrO_3_  -1Cu | 361 | 75 | 2014 | [29] |
|  | KNNS-Bi_0.5_(Na_0.82_K_0.18_)_0.5_ZrO_3_ | 427 | 52 | 2014 | [29] |
|  | KNN-1GdMnO_3_ | 97 | 1180 | 2016 | [30] |
|  | LKNNS9-Cu | 147 | 740 | 2016 | [31] |
|  | KNN-LS-0.8Bi(Fe_0.8_Co_0.2_)O_3_ | 199 | 58 | 2016 | [32] |
|  | LKNNS7-Cu | 241 | 56 | 2016 | [32] |
|  | KNN-LS-BiFeO_3_-Zn | 278 | 27 | 2016 | [33] |
|  | KNN-1.5CuF_2_ | 85 | 3170 | 2018 | [34] |
|  | KNN | 123 | 404 | 2018 | [35] |
|  | LKNNST | 180 | 60 | 2018 | [36] |
|  | LKNNT-CZ | 213 | 52 | 2018 | [37] |
|  | LKNNT-CZ-1Mn | 286 | 66 | 2018 | [37] |
|  | KNN-3.75(Bi_0.5_Na_0.5_)ZrO_3_  -BiScO_3_ | 352 | 57 | 2018 | [38] |
|  | KNNS-(Bi_0.5_Na_0.5_)_0.9_Co_0.1_ZrO_3_ | 430 | 15 | 2018 | [39] |
|  | KNNCu | 85 | 3110 | 2020 | [40] |
|  | KNN-2Mn | 109 | 330 | 2020 | [41] |
|  | KNNS-BiFeO_3_  -1(Bi_0.5_Na_0.5_)ZrO_3_ | 250 | 99 | 2020 | [42] |
|  | KNN-BZ-(Bi_0.5_Li_0.5_)TiO_3_  -0.375Zn | 355 | 66 | 2020 | [43] |
|  | KNNS-(Bi_0.5_Na_0.5_)ZrO_3_  -0.5BiFeO_3_ | 455 | 41 | 2020 | [44] |
|  | KNNS-BiFeO_3_  -3(Bi_0.5_Na_0.5_)ZrO_3_ | 550 | 51 | 2020 | [42] |
|  | KNNS-(Bi_0.5_Na_0.5_)ZrO_3_-1BZ | 610 | 34 | 2020 | [45] |

(If not specified, N = Na, N = Nb, L = Li, S = Sb, T = Ta, B = Ba, Z = Zr, C = Ca. The property values of PZT-based materials were derived from ref [11] and three companies in 2021. )

**Table S2.** *d*_33_ and *Q*_m_ of KNN-based materials with defect engineering

| Composition | *d*_33_ (pC/N) | *Q*_m_ | Reference |
| --- | --- | --- | --- |
| KNN-(Bi_0.5_K_0.5_)TiO_3_ | 165 | 85 | [46] |
| KNN-(Bi_0.5_K_0.5_)TiO_3_-0.4MnO | 197 | 115 |  |
| KNN-(Bi_0.5_K_0.5_)TiO_3_-0.8MnO | 221 | 162 |  |
| KNN | 64 | 108 | 47 |
| KNN-0.25K_4_CuNb_8_O_23_ | 95 | 1110 |  |
| KNN-0.5K_4_CuNb_8_O_23_ | 101 | 1503 |  |
| KNN | 90 | 78 | 48 |
| KNN-0.3Co_2_O_3_ | 122 | 124 |  |
| KNN-0.5 Co_2_O_3_ | 120 | 213 |  |
| KNN-SrZrO_3_ | 88 | 79 | 49 |
| KNN-SrZrO_3_-0.2ZrO_2_ | 104 | 87 |  |
| KNN-SrZrO_3_-0.4ZrO_2_ | 154 | 101 |  |
| KNN | 68 | 326 | 50 |
| KNNCu_0.25_O_3_ | 105 | 397 |  |
| KNNCu_0.5_O_3_ | 112 | 745 |  |
| LKNNS | 144 | 46 | 51 |
| LKNNSNi0.2 | 160 | 101 |  |
| LKNNSNi0.5 | 192 | 136 |  |
| LKNNT | 202 | 61 | 52 |
| LKNNT-0.2BiFeO_3_ | 212 | 70 |  |
| LKNNT-0.5BiFeO_3_ | 239 | 75 |  |
| KNNT-Bi_0.5_Na_0.5_ZrO_3_ | 317 | 66 | 53 |
| KNNT-Bi_0.5_Na_0.5_ZrO_3_-0.1MnO_2_ | 325 | 103 |  |
| KNNT-Bi_0.5_Na_0.5_ZrO_3_-0.2MnO_2_ | 330 | 135 |  |
| KNN-1Cu | 96 | 408 | 54 |
| KNN-1Cu-0.25SnO_2_ | 101 | 464 |  |
| KNN-1Cu-0.5SnO_2_ | 117 | 804 |  |
| KNN-1Cu-1SnO_2_ | 121 | 1038 |  |
| KNNS-KCT-LiCO_3_ | 151 | 808 | 55 |
| KNNS-KCT-LiCO_3_-0.3Bi_2_O_3_ | 183 | 1712 |  |
| KNN-0.01AlFeO_3_ | 88 | 80 | 56 |
| KNN-0.02AlFeO_3_ | 95 | 94 |  |
| KNN-0.03AlFeO_3_ | 117 | 115 |  |
| KNN | 110 | 60 | 57 |
| KNN-0.5MnO | 120 | 263 |  |
| KNN-1MnO | 112 | 313 |  |
| KNN | 89 | 407 | 58 |
| KNN-0.5GdMnO_3_ | 92 | 725 |  |
| KNN-1GdMnO_3_ | 97 | 1180 |  |
| KNN-LS | 216 | 26 | 59 |
| KNN-LS-Bi(Fe_0.8_Co_0.2)_O_3_ | 275 | 31 |  |

**References**

1. Saito Y, Takao H, Tani T *et al*. Lead-free piezoceramics. *Nature* 2004;**432**:84-7.

2. Wu JG, Xiao DQ, Wang YY *et al*. Effects of K/Na ratio on the phase structure and electrical properties of (K_x_Na_0.96-x_Li_0.04_)(Nb_0.91_Ta_0.05_Sb_0.04_)O_3_ lead-free ceramics. *Appl Phys Lett* 2007;**91**:252907.

3. Liang WF, Wu WJ, Xiao DQ *et al*. New crystallographic dielectric phase boundary in K_0.5_Na_0.5_NbO_3_-based lead-free ceramics. *Phys Status Solidi RRL* 2011;**5**:220-2.

4. Zhang BY, Wu JG, Cheng XJ *et al*. Lead-free piezoelectrics based on potassium-sodium niobate with giant *d*_33_. *ACS Appl Mater Inter* 2013;**5**:7718-25.

5. Wang XP, Wu JG, Xiao DQ *et al*. Giant piezoelectricity in potassium-sodium niobate lead-free ceramics. *J Am Chem Soc* 2014;**136**:2905-10.

6. Zheng T, Wu JG, Xiao DQ *et al*. Giant *d_33_* in nonstoichiometric (K, Na)NbO_3_-based lead-free ceramics. *Scripta Mater* 2015;**94**:25-7.

7. Xu K, Li J, Lv X *et al*. Superior piezoelectric properties in potassium-sodium niobate lead-free ceramics. *Adv Mater* 2016;**28**:8519-23.

8. Tao H, Wu HJ, Liu Y, *et al* Ultrahigh performance in lead-free piezoceramics utilizing a relaxor slush polar state with multiphase coexistence. *J Am Chem Soc* 2019;**141**:13987-94.

9. Go SH, Kim DS, Eum JM *et al*. Excellent piezoelectric properties of (K, Na)(Nb, Sb)O_3_-CaZrO_3_-(Bi, Ag)ZrO_3_ lead-free piezoceramics. *J Alloy Compd* 2021;**889**:161817.

10. Li P, Zhai JW, Shen B *et al*. Ultrahigh piezoelectric properties in textured (K, Na)NbO_3_‐based lead-free ceramics. *Adv Mater* 2018;**30**:1705171.

11. Li Z, Thong HC, Zhang YF *et al*. Defect engineering in lead zirconate titanate ferroelectric ceramic for enhanced electromechanical transducer efficiency. *Adv Funct Mater* 2021;**31**:2005012.

12. Chandrasekaran A, Damjanovic D, Setter N et al. Defect ordering and defect–domain-wall interactions in PbTiO_3_: A first-principles study. *Phys Rev B* 2013;**88**: 214116.

13. Li E, Kakemoto H, Hoshina T et al. A shear-mode ultrasonic motor using potassium sodium niobate-based ceramics with high mechanical quality factor. *Jpn J Appl Phys* 2008;**47**:7702-6.

14. Lee L and Yoo J. Piezoelectric and dielectric properties of low temperature sintering (K_0.5_Na_0.5_)NbO_3_ ceramics according to sintering aid Li_2_CO_3_. *J Korean Inst Electr Electron Mater Eng* 2008;**21**:906-10.

15. Park HY, Seo IT, Choi MK *et al*. Microstructure and piezoelectric properties of the CuO-added (Na_0.5_K_0.5_)(Nb_0.97_Sb_0.03_)O_3_ lead-free piezoelectric ceramics. *Journal of Applied Physics* 2008;**104**:034103.

16. Lee Ii, Yoo J and Jeong Y. Piezoelectric and dielectric properties of low temperature sintering (K_0.5_Na_0.5_)NbO_3_ ceramics with the variation of poling electric field. *J Korean Inst Electr Electron Mater Eng* 2008;**21**:1000-4.

17. Park BC, Hong IK, Jang HD *et al*. Highly enhanced mechanical quality factor in lead-free (K_0.5_Na_0.5_)NbO_3_ piezoelectric ceramics by co-doping with K_5.4_Cu_1.3_Ta_10_O_29_ and CuO. *Mater Lett* 2010;**64**:1577-79.

18. Cha YJ, Jeong YH, Lee YJ *et al*. Structural and piezoelectric properties of (1- *x*)(Na_0.5_K_0.5_)NbO_3_-*x*Ba(Ti_0.9_Sn_0.1_)O_3_ lead-free ceramics. *Jpn J Appl Phys* 2010;**49**:020205.

19. Jiang XP, Chen Y, Lam KH *et al*. Effects of MnO doping on properties of 0.97K_0.5_Na_0.5_NbO_3_-0.03(Bi_0.5_K_0.5_)TiO_3_ piezoelectric ceramics. *J Alloy Compd* 2010;**506**:323-6.

20. Tai WP, Ji HN, Ok YP *et al*. Preparation of lead-free (K, Na)NbO_3_-LiSbO_3_ ceramics with high piezoelectric constant by FeO doping. *J Korean Phy Soc* 2010;**56**:1156-9.

21. Yang SL, Tsai CC, Liou YC *et al*. Differences between copper-oxide- and zinc-oxide-doped sodium potassium niobate ceramics. Romero J, ed. *J Am Ceram Soc* 2012;**95**:2110-2.

22. Li HT, Zhang BP, Li Q *et al*. Phase and electrical properties of [Li_0.065_(Na_0.535_K_0.48_)_0.95_]NbO_3_ lead-free piezoelectric ceramics sintered at low temperature. *Adv Mat Res* 2012;**415-417**:1679-82.

23. Ariizumi T, Zushi J, Kojima S *et al*. Effects of Mn additive on dielectric and piezoelectric properties of (Na_0.5_K_0.5_)NbO_3_-BaZrO_3_-(Bi_0.5_K_0.5_)TiO_3_ ternary system. *Jpn J Appl Phys* 2012;**51**:07GC01.

24. Liang WF, Xiao DQ, Wu JG *et al*. Origin of high mechanical quality factor in CuO-doped (K, Na)NbO_3_-based ceramics. *Front Mater Sci* 2014;**8**:165-75.

25. Liu C, Xiao DQ, Wu JG *et al*. Electrical properties of CuO-doped (K_0.5_Na_0.5_)(Nb_0.92_Sb_0.03_Ta _0.05_)O_3_ piezoelectric ceramics with high *Q*_m_. *Ferroelectrics* 2014;**458**:31-6.

26. Yin N, Jalalian A, Gai ZG *et al*. Effect of doping ions on structure and electrical properties of lead-free KNN ceramics. *Adv Mat Res* 2014;**1058**:190-5.

27. Li YM, Shen ZY, Wu F *et al*. Enhancement of piezoelectric properties and temperature stability by forming an MPB in KNN-based lead-free ceramics. *J Mater Sci: Mater Electron* 2014;**25**:1028-32.

28. Zhao XY, Wang H, Xu JW *et al*. Microstructure and electrical properties of K_0.5_Na_0.5_NbO_3_-LiSbO_3_-BiFeO_3_-*x*%molZnO lead-free piezoelectric ceramics. *Journal of Elec Materi* 2014;**43**:506-11.

29. Wu JG. High piezoelectricity in low-temperature sintering potassium-sodium niobate-based lead-free ceramics. *RSC Adv* 2014;**4**:53490-7.

30. Bucur RA, Badea I, Bucur AI *et al*. Good quality factor in GdMnO_3_-doped (K_0.5_Na_0.5_)NbO_3_ piezoelectric ceramics. *J Electron Mater* 2016;**45**:3046-52.

31. López-Juárez R, González-García F and Villafuerte-Castrejón ME. Effects of CuO doping on the piezoelectric properties of KNLNS-BZ ceramics. *J Mater Sci: Mater Electron* 2016;**27**:7369-73.

32. Wang H, Zhao XY, Xu JW *et al*. Structure and properties of (1-x)[(K_0.5_Na_0.5_)NbO_3_-LiSbO_3_]-*x*BiFe_0.8_Co_0.2_O_3_ lead-free piezoelectric ceramics. *Bull Mater Sci* 2016;**39**:743-7.

33. Wang H, Zhao XY, Xu JW *et al*. Effects of sintering temperature on structure and properties of 0.98[K_0.5_Na_0.5_NbO_3_-LiSbO_3_-BiFeO_3_]-0.02ZnO piezoelectric ceramics. *J Mater Sci: Mater Electron* 2016;**27**:2036-41.

34. Weng CM, Tsai CC, Hong CS *et al*. Effects of post-annealing on electrical properties of CuF_2_·*x*H_2_O-doped KNN ceramics for rotary-linear ultrasonic motors. *Ceram Int* 2018;**44**:16173-80.

35. Nandini RN, Krishna M, Suresh AV *et al*. Effect of MWCNTs on piezoelectric and ferroelectric properties of KNN composites. *Mat Sci Eng B-Adv* 2018;**231**:40-56.

36. Zheng MP, Hou YD, Chao LM *et al*. Piezoelectric KNN ceramic for energy harvesting from mechanochemically activated precursors. *J Mater Sci: Mater Electron* 2018;**29**:9582-7.

37. Yao FZ, Zhang MH, Wang K *et al*. Refreshing piezoelectrics: distinctive role of manganese in lead-free perovskites. *ACS Appl Mater Inter* 2018;**10**:37298-306.

38. Jiang LM, Tan Z, Xie LX *et al*. Study of the relationships among the crystal structure, phase transition behavior and macroscopic properties of modified (K,Na)NbO_3_ -based lead-free piezoceramics. *J Eur Ceram Soc* 2018;**38**:2335-43.

39. Xie LX, Xing J, Tan Z *et al*. Comprehensive investigation of structural and electrical properties of (Bi, Na)CoZrO_3_-doped KNN ceramics. *J Alloy Compd* 2018;758:14-24.

40. Liao Y, Wang DM, Wang H *et al*. Modulation of defects and electrical behaviors of Cu-doped KNN ceramics by fluorine-oxygen substitution. *Dalton Trans* 2020;**49**:1311-8.

41. Xu Z, Lou LY, Zhao CL *et al*. Effect of manganese doping on ferroelectric and piezoelectric properties of KNbO_3_ and (K_0.5_Na_0.5_)NbO_3_ lead-free ceramics. *Acta Phys Sin* 2020;**69**:127705.

42. Yao WZ, Zhang JL, Zhou CM *et al*. Giant piezoelectricity, rhombohedral-orthorhombic-tetragonal phase coexistence and domain configurations of (K, Na)(Nb, Sb)O_3_-BiFeO_3_-(Bi, Na)ZrO_3_ ceramics. *J Eur Ceram Soc* 2020;**40**:1223-31.

43. Li JW, Liu YX, Thong HC *et al*. Effect of ZnO doping on (K, Na)NbO_3_-based lead-free piezoceramics: Enhanced ferroelectric and piezoelectric performance. *J Alloy Compd* 2020;**847**:155936.

44. Wu B, Ma J, Wu WJ *et al*. Improved piezoelectricity in ternary potassium-sodium niobate lead-free ceramics with large strain. *J Mater Chem C* 2020;**8**:2838-46.

45. Zhou CM, Zhang JL, Yao WZ *et al*. Remarkably strong piezoelectricity, rhombohedral-orthorhombic-tetragonal phase coexistence and domain structure of (K, Na)(Nb, Sb)O_3_-(Bi, Na)ZrO_3_-BaZrO_3_ ceramics. *J Alloy Compd* 2020;**820**:153411.

46 Jiang XP, Chen Y, Lam KH *et al*. Effects of MnO doping on properties of 0.97K_0.5_Na_0.5_NbO_3_-0.03(Bi_0.5_K_0.5_)TiO_3_ piezoelectric ceramics. *J Alloy Compd* 2010; **506**:323-326.

47 Lim JB, Zhang SJ, Jeon JH, *et al*. (K,Na)NbO_3_-based ceramics for piezoelectric “hard”lead-free materials. *J Am Ceram Soc* 2010:1218-1220.

48 Wu WJ, Xiao DQ, Wu JG *et al*. Phase structure, piezoelectric and multiferrioc behavior of (K_0.48_Na_0.52_)NbO_3_-Co_2_O_3_ piezoelectric ceramics. *Funct Mater Lett* 2012; 04:225-229.

49 Kang KM, Cho JH, Nam JH *et al*. Sintering of ZrO_2_-modified 0.96(K_0.5_Na_0.5_)NbO_3_-0.04SrZrO_3_ piezoelectric ceramics in a reduced atmosphere. *J Korean Inst Electr Electron Mater Eng* 2011; 24:563-567.

50 Tan X, Fan H, Ke S *et a*l. Structural dependence of piezoelectric, dielectric and ferroelectric properties of K_0.5_Na_0.5_(Nb_1−2x/5_Cu_x_)O_3_ lead-free ceramics with high *Q*_m_. *Mater Res Bull* 2012; 47:4472-4477.

51 Shen ZY, Li YM, Luo WQ, *et al*. Enhancing piezoelectric properties by introducing Ni ion into B–site of Li/Sb-modified (K,Na)NbO_3_ Pb-free piezoceramics. *J Ceram Soc Jpn* 2012; 120:375-377.

52 Zhou JJ, Li JF, Cheng LQ *et al*. Addition of small amounts of BiFeO_3_ to (Li,K,Na)(Nb,Ta)O_3_ lead-free ceramics: Influence on phase structure, microstructure and piezoelectric properties. *J Eur Ceram Soc* 2012; 32:3575-3582.

53 Luo W. Effect of MnO₂ on the electrical properties of 0.96KNNT-0.04BNZ lead-free piezoelectric ceramics. *Ceram-Silikaty* 2019:399-402.

54 Su S, Zuo RZ, Wang XH, Li LT. Sintering, microstructure and piezoelectric properties of CuO and SnO_2_ co-modified sodium potassium niobate ceramics. *Mater Res Bull* 2010; 45:124-128.

55 Noh J, Yoo J. Dielectric and piezoelectric properties of (K_0.5_Na_0.5_)(Nb_0.97_Sb0_.03_)O_3_ ceramics doped with Bi_2_O_3_. *J Electroceram* 2012; 29:144-148.

56 Ling P, Minhong J, Zheng FG *et a*l. Structure and piezoelectric properties of K_0.5_Na_0.5_NbO_3_-AlFeO_3_ lead-free ceramics by using AlFeO_3_ as a sintering aid. J Mater Sci-Mater El 2013; 25:323-327.

57 Lopez-Juarez R, Gomez-Vidales V, Cruz MP, *et al*. Dielectric, ferroelectric, and piezoelectric properties of Mn-doped K_0.5_Na_0.5_NbO_3_ lead-free ceramics. *J Electron Mate*r 2015; 44:2862-2868.

58 Bucur RA, Badea I, Bucur AI, *et al*. Good quality factor in GdMnO_3_-doped (K_0.5_Na_0.5_)NbO_3_ piezoelectric ceramics. *J Electron Mate*r 2016; 45:3046-3052.

59 Wang H, Zhao X, Xu J *et al*. Structure and properties of (1-x)[(K_0.5_Na_0.5_)NbO_3_-LiSbO_3_]- xBiFe_0.8_Co_0.2_O_3_ lead-free piezoelectric ceramics. B Mater Sci 2016; 39:743-747.
